# Supplementary material for: Evidence supported by Mendelian randomization: impact on inflammatory factors in knee osteoarthritis
Source: Front Med (Lausanne). 2024 May 28;11:1382836. doi: 10.3389/fmed.2024.1382836 (PMC11165061; doi:10.3389/fmed.2024.1382836)
Supplement: Supplementary file 1 [file Data_Sheet_1.ZIP › data/GCST90274772_5e6/772.forest.pdf]

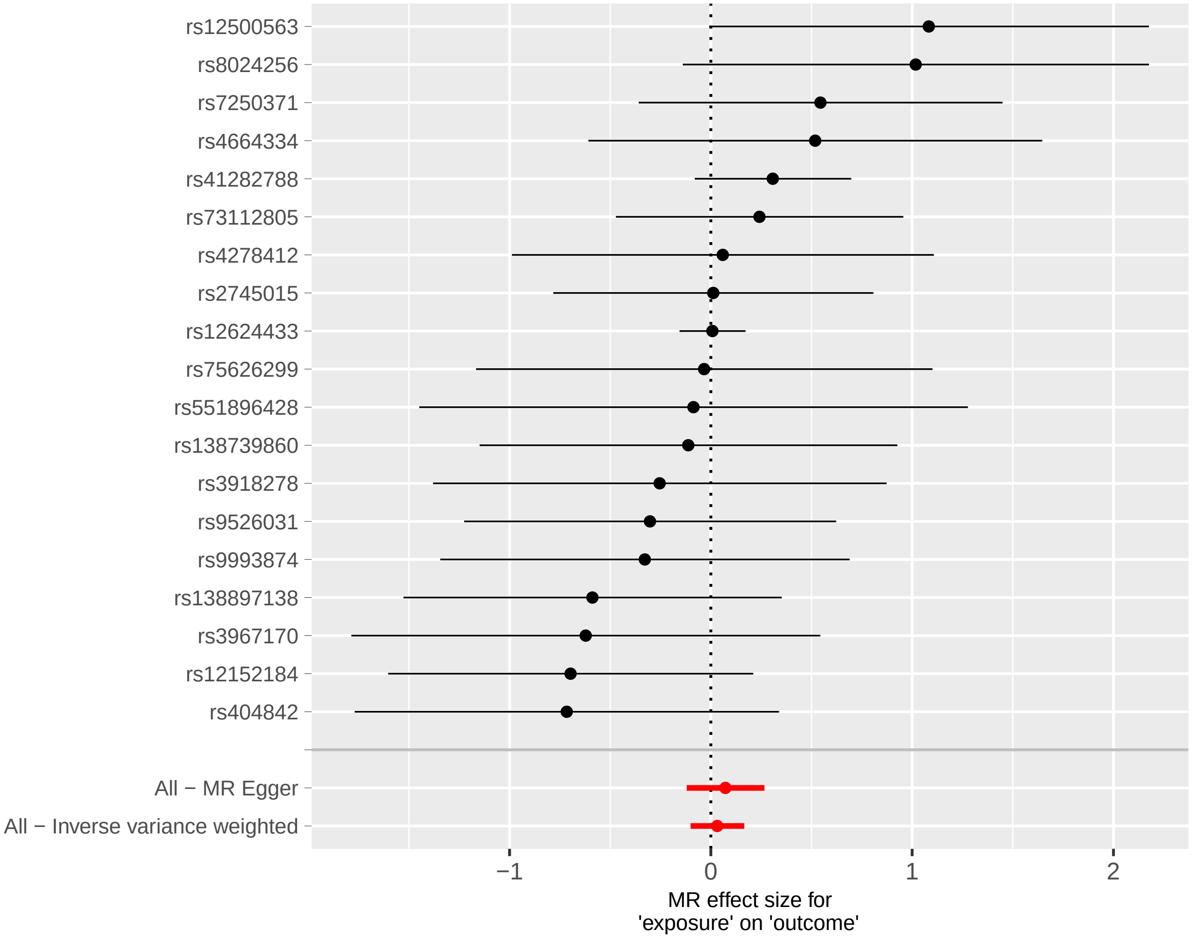
rs12500563
rs8024256
rs7250371
rs4664334
rs41282788
rs73112805
rs4278412
rs2745015
rs12624433
rs75626299
rs551896428
rs138739860
rs3918278
rs9526031
rs9993874
rs138897138
rs3967170
rs12152184
rs404842
All − MR Egger
All − Inverse variance weighted
−1
0
1
2
MR effect size for
'exposure' on 'outcome'
